# Lipopolysaccharide Structural Differences between Western and Asian *Helicobacter pylori* Strains

**DOI:** 10.3390/toxins10090364

**Published:** 2018-09-08

**Authors:** Hong Li, Hong Tang, Aleksandra W. Debowski, Keith A. Stubbs, Barry J. Marshall, Mohammed Benghezal

**Affiliations:** 1 West China Marshall Research Center for Infectious Diseases, Center of Infectious Diseases, West China Hospital of Sichuan University, Chengdu 610041, China; hong.li@cd120.com; 2 *Helicobacter pylori* Research Laboratory, School of Biomedical Sciences, Marshall Centre for Infectious Disease Research and Training, University of Western Australia, Nedlands, WA 6009, Australia; aleksandra.debowski@uwa.edu.au (A.W.D.); barry.marshall@uwa.edu.au (B.J.M.); 3 School of Molecular Sciences, University of Western Australia, Crawley, WA 6009, Australia; keith.stubbs@uwa.edu.au

**Keywords:** *Helicobacter pylori*, lipopolysaccharide, structure

## Abstract

Recent structural analysis of the lipopolysaccharide (LPS) isolated from *Helicobacter pylori* G27 wild-type and *O*-antigen ligase mutant resulted in the redefinition of the core-oligosaccharide and *O*-antigen domains. The short core-oligosaccharide (Glc–Gal–Hep-III–Hep-II–Hep-I–KDO) and its attached trisaccharide (Trio, GlcNAc–Fuc–Hep) appear to be highly conserved structures among *H. pylori* strains. The G27 LPS contains a linear glucan–heptan linker between the core-Trio and distal Lewis antigens. This linker domain was commonly identified in Western strains. In contrast, out of 12 partial LPS structures of Asian strains, none displayed the heptan moiety, despite the presence of Lewis antigens. This raises the question of how Lewis antigens are attached to the Trio, and whether the LPS structure of Asian strains contain another linker. Of note, a riban was identified as a linker in LPS of the mouse-adapted SS1 strain, suggesting that alternative linker structures can occur. In summary, additional full structural analyses of LPS in Asian strains are required to assess the presence or absence of an alternative linker in these strains. It will also be interesting to study the glucan-heptan linker moieties in pathogenesis as *H. pylori* infections in Asia are usually more symptomatic than the ones presented in the Western world.

## 1. Introduction

*Helicobacter pylori* infection is an infectious disease affecting more than half of the world’s population [1]. The infection is primarily acquired in childhood and presents for decades without combination antibiotic treatment. The chronic infection results in active gastritis in all colonised subjects, which can ultimately lead to the development of gastric adenocarcinoma [2].

The cell wall of *H. pylori*, like most other Gram-negative bacteria, contains a complex glycolipid structure, lipopolysaccharide (LPS), which is actively involved in interactions between the bacterium and its host. Conceptually, *H. pylori* LPS structure is divided into three parts: lipid A, a hydrophobic domain anchored in the cell wall; the core-oligosaccharide domain, and the outermost *O*-antigen domain [3]. Through enzymatc dephosporylation and deacylation, the *de novo* synthesised lipid A in *H. pylori* is constitutively modified into an unusual lipid A with the loss of nagative charges, which endows *H. pylori* with intrinsic resisance to antimicrobial peptides and the evasion of host innate immune system [4]. The enzymes involved in the *de novo* biosynthesis and constitutve modification of *H. pyori* lipid A have been identified [3]. Several studies have demonstrated that the *H. pylori* lipid A structures are conserved among strains isolated from different geographical regions [5,6,7,8,9,10,11,12,13,14]. Due to the fact that the glycosyltransferase genes responsible for the *H. pylori* LPS biosynthesis are dispersed across the genome, many *H. pylori* LPS glycosyltransferases remain to be identified [15]. In addition, structural studies on *H. pylori* LPS have demonstrated variations in the polysaccharide region of LPS from different strains [5,6,7,8,9,10,11,12,13,14].

*H. pylori* infections in Asian countries are more symptomatic and severe than the ones presented in the Western world [16]. Given the fact that LPS is a major constituent of the OM, this review provides an overview of the current knowledge about the LPS structures of Western and Asian strains which could be ultimately used to assess whether LPS structural differences contribute to the different clinical outcomes. In addition, the glycosyltransferases involved in *H. pylori* LPS biosynthesis are also summarised.

## 2. LPS Structure in Western *H. pylori* Strains

### 2.1. LPS Structure and Biosynthesis in H. pylori Strains 26695 and G27

**Figure 1:**
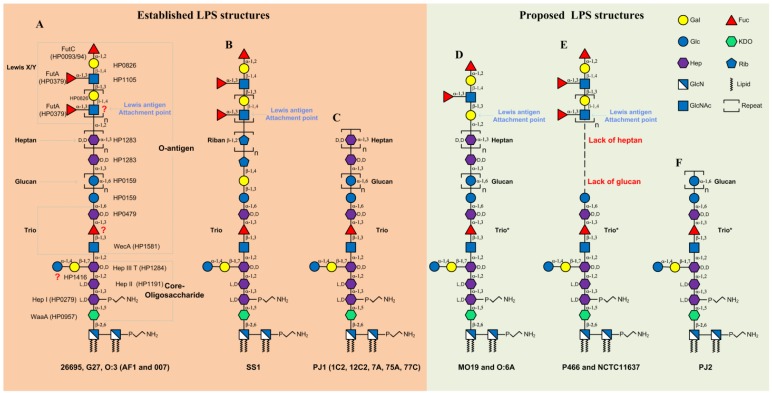
Established and proposed lipopolysaccharide (LPS) structures in Western *H. pylori* strains. Only three established LPS structures are available (**A**–**C**). Assuming the Trio is conservatively present in *H. pylori* LPS, 3 additional LPS structural models can be proposed from partial structures reported in the literature (**D**–**F**). (**A**) LPS structures in strains 26695 [11], G27 [9], O:3 [5,10], AF1, and 007 [19]. The general architecture of the LPS structures in these strains are very similar, although the length of the glucan and heptan, the fucosylation, and number of repeats of the Lewis chains, vary between strains. The *O*-antigen is defined to encompass the Trio, the glucan-heptan linker and the Lewis antigens. The Lewis antigen attachment point is suggested to be a GlcNAc residue. Previously known *H. pylori* LPS biosynthetic enzymes are presented, whereas the unidentified enzymes are indicated by red question marks; (**B**) LPS structure in strain SS1 [12]. No glucan (however a single Glc residue is present after the Trio) and no heptan are present, instead, a rare riban structure (oligomer of Rib) is inserted between the core-Trio and Lewis antigen structures. The Lewis antigen is connected to the riban through a GlcNAc residue; (**C**) LPS structures in strains PJ1, 1C2, 12C2, 7A, 75A, and 77C [20]. LPS in these strains contains the glucan and heptan, but lacks the Lewis antigens; (**D**) LPS structures in strains MO19 and O:6A [5,7]. The glucan and heptan structures are present, whereas only a single Le^y^ unit is detected in the two strains. The Lewis antigen is attached to the heptan via a Gal residue rather than the GlcNAc residue; (**E**) LPS structures in strains P466 [7] and NCTC11637 [14]. The glucan and heptan structures are absent in these two strains; (**F**) LPS structures in strain PJ2 ends with the glucan but lacks the heptan and Lewis antigens. * The Trio structure is inferred to be conservatively present in these strains.

Collected from an English patient, 26695 is the first fully sequenced *H. pylori* strain in 1997 [17]. The G27 strain was collected from an Italian patient and also fully sequenced in 2009 [18]. The LPS structures from strains 26695 and G27 are currently the most-studied and best-characterized [9,11]. Figure 1A summarises the latest data of the structure of 26695 and G27 LPS as well as the corresponding biosynthetic glycosyltransferases.

Early structural studies proposed the *O*-antigen of 26695 LPS comprising the Lewis antigen only, and the core-oligosaccharide domain of 26695 LPS encompassing an inner core and outer core [6,21,22,23,24]. The inner core was defined as a hexasaccharide (Glc–Gal–Hep-III–Hep-II–Hep-I–KDO) whereas the outer core was defined as a DD–heptan and a lateral-branched α-1,6-glucan [6,21,22,23,24]. However, later reinvestigation of the structure of LPS from 26695 revealed a newly identified trisaccharide motif (Trio, GlcNAc–Fuc–Hep) followed by the linear arrangement of the glucan and heptan [11]. Very recently, through amplication of mass spectrometry and NMR techniques, our group analysed the LPS structures of the G27 wild-type and its isogenic *O*-antigen ligase mutant Δ*waaL* [9]. Our data showed that the LPS structures in strains G27 and 26695 are almost identical [9]. Of note, the core-oligosaccharide structure in the Δ*waaL* mutant was shown to be composed solely of the hexasaccharide, indicating that the Trio, the linear glucan-heptan, and the Lewis antigen are all transferred by the *O*-antigen ligase (Figure 1A). This finding allowed for a reassessment of the previous *H. pylori* LPS model and resulted in the redefined *H. pylori* LPS *O*-antigen domain encompassing not only the Lewis antigen, but also the Trio and the glucan-heptan linker; and the redefined core-oligosaccharide domain encompassing the short hexasaccharide only [9].

The three Hep residues of the core-oligosaccharide (Glc–Gal–Hep-III–Hep-II–Hep-I–KDO) are known to be transferred by Hep I transferase (HP0279), Hep II transferase (HP1191) and Hep III transferase (HP1284), respectively [15,25]. As for the assembly of the branched disaccharide Glc-Gal linking to the Hep-III, the Glc transferase is proposed to be encoded by *HP1416* [26,27], whereas the Gal transferase has yet to be identified. Based on the newly defined *H. pylori O*-antigen, the GlcNAc residue of the Trio structure (Figure 1A), is assembled by the *O*-antigen initiating enzyme WecA (HP1581) [28], allowing *O*-antigen elongation and subsequent ligation onto the core-oligosaccharide via GlcNAc. The Hep residue of the Trio is transferred by HP0479 [11,24], whereas the Trio fucosyltransferase remains to be identified. Following the Trio is the α-1,6-glucan structure, which is known to be assembled by the glucosyltransferase HP0159 [22,26,27]. The heptosyltransferase responsible for assembling the heptan structure is inferred to be encoded by *HP1283*, based on a very recent observation that the mutation of *HP1283* homolog in strain PJ1 resulted into the loss of the heptan structure [29].

In the LPS of 26695 and G27 (Figure 1A), the heptan is capped by a long type 2 Le^x/y^ chain [9,11]. No type 1 Le^a^ and Le^b^ antigens have been detected in these two strains. A Gal-β-(1,4)-GlcNAc linkage comprises the type 2 Lewis LacNAc backbone, in which the addition of Gal to GlcNAc is transferred by the β-(1,4)-Gal transferase HP0826 [11,21], whereas the β-(1,3)-GlcNAc transferase HP1105 is responsible for adding GlcNAc to Gal for the LacNAc elongation [23]. Of note, the glycosyltransferase adding the first GlcNAc to the heptan for the LacNAc initiation has yet to be identified. Three fucosyltransferases (FucTs): HP0379 (FutA), HP0651 (FutB), and HP0093/94 (FutC), have been identified to be responsible for the fucosylation of the polyLacNAc to generate the Le^x^ and Le^y^ antigens [30,31].

All the currently known glycosyltransferases involved in 26695 LPS core-oligosaccharide and *O*-antigen biosynthesis are summarised in Table 1.

### 2.2. LPS Structure in Other Western H. pylori Strains

In addition to the above well-characterised LPS structures in 26695 and G27, partial LPS structural analyses have also been conducted in other Western *H. pylori* strains including NCTC11637, SS1, O:3, O:6, MO19, P466, PJ1, PJ2, and the Danish strains AF1 and 007 [5,6,7,10,12,19,29,37]. Of note, is that the short core-oligosaccharide Glc–Gal–Hep-III–Hep-II–Hep-I–KDO has been demonstrated to be conserved in the LPS of all strains examined to date [5,6,7,8,9,10,11,12,13,14,29,37]. The Trio has also been detected in the LPS structures of other *H. pylori* strains, such as O:3 (Figure 1A) [10], SS1 (Figure 1B) [12] and PJ1 (Figure 1C) [29], and our laboratory also identified the trio in the LPS of strain X47 [9]. This suggests that the Trio, the beginning of the *O*-antigen, is conserved in *H. pylori*. Interestingly, this Trio moiety was not detected in previously analysed structures of LPS from 26695, SS1, O:3, and PJ1 [5,6,20,37]. This may be ascribed to the difficulty of LPS structural analysis and less developed techniques available at the time. Thus, only three different full LPS structures are available (Figure 1A–C). Assuming that the Trio is a conserved motif in the LPS of *H. pylori*, three additional LPS structural models have been built from previous publications of the partial structures (Figure 1D–F).

LPS structures in MO19, O:3, O:6 [5,7], and the Danish strains AF1 and 007 [19] are very similar to that of LPS in 26695 and G27 (Figure 1A). The glucan–heptan and the outermost type 2 Le^x/y^ antigens are present in LPS from strains MO19, O:3, O:6A [5,7] (Figure 1D). However, in these strains, the attachment point for the Lewis antigens to the heptan is via a Gal instead of a GlcNAc residue as in 26695, G27, O:3, AF1, and 007 strains (compare Figure 1D with Figure 1A). In the SS1 strain, the linker between the core-Trio and the distal Lewis antigen is a rare riban structure (oligomer of ribofuranose) instead of the glucan–heptan [12] (Figure 1B). In asymptomatic strains (Hp1C2, Hp7A, Hp12C2, Hp75A, Hp77C, and PJ1), the glucan–heptan is present, whereas the Lewis antigens are missing (Figure 1C) [20]. The lack of the glucan–heptan linker was observed in the LPS of strains NCTC11637 [14] and P466 [7], nevertheless, the outermost Lewis antigens remain present (Figure 1E). Strains UA948, UA955, and J223 also lack the glucan–heptan linker, but simultaneously express both type 1 and type 2 Lewis antigens [13]. In strain PJ2, all the distal heptan and Lewis antigens are absent (Figure 1F) [37].

It has been reported that the LPS glucan is expressed by the majority of *H. pylori* isolates from Greek children [38]. Another study by the same group reported the glucan expression was 73% (30/41) in Canadian *H. pylori* isolates [39]. Data from carbohydrate analysis performed directly on bacterial cells in the above two studies show the majority of the glucan-positive strains simultaneously express heptan [38,39]. Therefore, it seems likely that the glucan–heptan linker is commonly expressed in LPS of Western *H. pylori* strains.

The type 2 Le^x/y^ antigens have been demonstrated to be expressed in 80–90% of both Western and Asian *H. pylori* strains [3]. However, it should be noted that the type 1 Le^a/b^ antigen expression is also common in Western *H. pylori* isolates [38,39,40]. Recently, it has been demonstrated that the type 1 Le^b^ antigen expression is around 20% in Chilean, Greek, and Canadian clinical isolates [38,39,40]. Structural analysis studies demonstrated that type 1 and type 2 structural regions can be simultaneously expressed in the same *O*-chain molecule [8,13]. For example, the type 1 Le^a^ is found to be a terminal structure that caps the type 2 Le^x^ chains in Canadian strain UA948; the type 1 chain precursor Le^c^ (Gal-β-(1,3)-GlcNAc) and Le^d^ (Fuc-α-(1,2)-Gal-β-(1,3)-GlcNAc) are found to be terminal structures that cap type 2 poly-LacNAc chains in strain J223 [13].

## 3. LPS Structures in Asian *H. pylori* Strains

**Figure 2:**
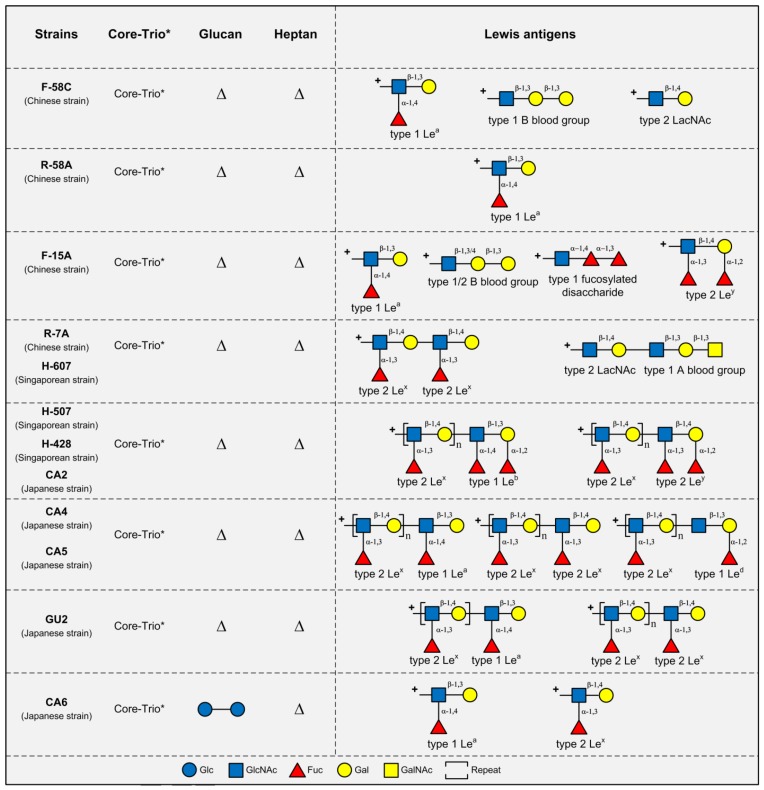
LPS structures in 12 Asian *H. pylori* strains. LPS structures in 4 Chinese strains (F-58C, R-58A, F-15A, and R-7A), 3 Singaporean strains (H-607, H-507, and H-428), and 5 Japanese strains (CA2, CA4, CA5, GU2, and CA6) [8]. * All these strains are assumed to have the same conserved core-Trio structures. ∆ means the structure is absent. The heptan is completely absent in the LPS structures of all 12 Asian strains, which is accompanied by the simultaneous absence of the glucan (with CA6 LPS as an exception, it has a glucan composed of two Glc residues). However, for strain R-58A, LPS in all the Asian strains have simultaneous expression of type 1 and type 2 Lewis chains. Strikingly, the type 1 Lewis chain (Gal-β-(1,3)-GlcNAc) can serve as a capping structure connected to type 2 chain (Gal-β-(1,4)-GlcNAc) in a single LPS molecule. For example, the LPS molecule in strains H-507, H-428, and CA2 is capped by a type 1 Le^b^ connected to polymeric type 2 Le^x^ chain. **+** indicates the Lewis antigen attachment point to the core-Trio structures.

LPS structural analyses have been conducted in 4 Chinese strains (F-58C, R-58A, F-15A, and R-7A), 5 Japanese strains (CA2, CA4, CA5, CA6, and GU2) and 3 Singaporean strains (H-507, H-607, and H-428) (Figure 2) [8]. Like the LPS of Western strains, the majority of these strains (10/12) express the type 2 Le^x/y^ antigens. However, compared with the LPS from Western strains, the LPS from these Asian strains tended to co-produce type 1 chains. LPS from strains F-58C and R-58A carry type 1 Le^a^ without the presence of type 2 Le^x/y^ antigens, whereas the remaining 10 strains had concurrent expression of both type 1 and type 2 chains. It was shown that type 1 Le^a/b/d^ could be displayed as terminal epitopes to cap the type 2 chains in strains CA2, CA4, CA5, GU2, H-428, and H-507 [8].

Interestingly, the most striking LPS structural difference between the Western and Asian *H. pylori* strains is the absence of the heptan domain, and the near complete absence of glucan (except in LPS of strain CA6 where two Glc units were found) in the LPS of all 12 of the above Asian strains [8].

As expected, LPS structures from these 12 Asian strains were found to contain the conserved core-oligosaccharide region. Although the Trio motif was not reported in the LPS structures of these Asian strains, there is genetic evidence for the presence of the Trio in these strains: the two genes *wecA* (*HP1581*) and *HP0479*, involved in the assembly of the Trio GlcNAc and Hep residues, respectively, are highly conserved in *H. pylori* genomes [3]. The failure in detection of the Trio in the LPS of these Asian strains is likely due to the technical challenges in LPS structural analysis at the time of study. Thus, we propose that the Trio, together with the redefined core-oligosaccharide, are highly conserved LPS domains in all *H. pylori* strains either from Western or Asian origins. The presence of a linker between the Trio and the commonly expressed Lewis antigen remains to be investigated in the LPS from Asian strains.

## 4. Conclusions and Future Directions

In summary, recent studies have provided new insights into LPS structural organisation in *H. pylori* [9,10,11,12]. The core-oligosaccharide domain of *H. pylori* LPS has been redefined as a short hexasaccharide [9,15]. The *O*-antigen in strains 26695 and G27 has been redefined, and bears a linear arrangement starting from the Trio, followed by the glucan-heptan linker, and the Lewis antigens [9]. The Trio appears to be conservatively present in *H. pylori* LPS structures, whereas the linker between the core-oligosaccharide domain and the commonly expressed Lewis antigens is not.

The most striking LPS structural difference between Western and Asian *H. pylori* strains is the presence and absence of the glucan–heptan linker, respectively. An alternative rare linker structure in the mouse adapted SS1 strain highlights the ability of *H. pylori* to shape its LPS structure, suggesting a role of the linker domain in adaptation to the host. The presence of the riban also shows *H. pylori*’s genetic plasticity to generate glycosyltransferases with new specificities. It has been suggested that the presence of the heptan may serve as a biological arm to increase the LPS length and flexibility, thus presenting Lewis antigens more easily for molecular mimicry to escape host immune detection [20]. Why the Western *H. pylori* strains commonly integrate a significant DD–heptan into their LPS structures is intriguing, since the DD–Hep residue is rarely encountered as a component of bacterial LPS molecules. If the function of the heptan is, as suggested, merely for increasing the LPS length [20], then why is heptan the building block, and not other carbohydrate motifs? The exact roles of the heptan structure in *H. pylori* pathogenesis warrant further genetic, immunologic, and in vivo studies.

Future structural and genetic studies of LPS from *H. pylori* Asian strains are needed to analyse whether all Asian strains lack the heptan region or have evolved another linker domain, and thus, identification of the corresponding biosynthetic genes in both Western and Asian strains will also be needed.

## Figures and Tables

**Table 1:** Currently known glycosyltransferase genes involved in 26695 LPS core-oligosaccharide and *O*-antigen biosynthesis.

GT Genes	Proposed/Demonstrated Functions	References
*HP0279*	Hep I transferase, assembling the core-oligosaccharide	[24,32]
*HP1191*	Hep II transferase, assembling the core-oligosaccharide	[25,33]
*HP1284*	Hep III transferase, assembling the core-oligosaccharide	[15]
*HP1416*	Glc transferase, assembling the core-oligosaccharide	[26,27]
*HP1581*	GlcNAc transferase (WecA), initiating the *O*-antigen assembly	[15,28]
*HP0479*	Hep transferase, assembling the Trio motif	[24]
*HP0159*	Glc transferase, assembling the glucan structure	[22,26,27]
*HP1283*	Hep transferase, assembling the heptan structure	[29]
*HP0826*	β(1,4)Gal transferase, assembling the Lewis chain	[11,21]
*HP1105*	β(1,3)GlcNAc transferase, assembling the Lewis chain	[23]
*HP0379*	FutA, α(1,3/4)Fuc transferase, assembling the Lewis chain	[30,34,35]
*HP0651*	FutB, α(1,3/4)Fuc transferase, assembling the Lewis chain	[30,34,35]
*HP0093/94*	FutC, α(1,2)Fuc transferase, assembling the Lewis chain	[31,34,35,36]
